# Surgical instrument detection and tracking technologies: Automating dataset labeling for surgical skill assessment

**DOI:** 10.3389/frobt.2022.1030846

**Published:** 2022-11-04

**Authors:** Shubhangi Nema, Leena Vachhani

**Affiliations:** Systems and Control Group, Indian Institute of Technology, Mumbai, Maharashtra, India

**Keywords:** MIS, surgical instrument detection and tracking, surgical skill assessment, dataset labeling, AI/ML

## Abstract

Surgical skills can be improved by continuous surgical training and feedback, thus reducing adverse outcomes while performing an intervention. With the advent of new technologies, researchers now have the tools to analyze surgical instrument motion to differentiate surgeons’ levels of technical skill. Surgical skills assessment is time-consuming and prone to subjective interpretation. The surgical instrument detection and tracking algorithm analyzes the image captured by the surgical robotic endoscope and extracts the movement and orientation information of a surgical instrument to provide surgical navigation. This information can be used to label raw surgical video datasets that are used to form an action space for surgical skill analysis. Instrument detection and tracking is a challenging problem in MIS, including robot-assisted surgeries, but vision-based approaches provide promising solutions with minimal hardware integration requirements. This study offers an overview of the developments of assessment systems for surgical intervention analysis. The purpose of this study is to identify the research gap and make a leap in developing technology to automate the incorporation of new surgical skills. A prime factor in automating the learning is to create datasets with minimal manual intervention from raw surgical videos. This review encapsulates the current trends in artificial intelligence (AI) based visual detection and tracking technologies for surgical instruments and their application for surgical skill assessment.

## 1 Introduction

**FIGURE 1:**
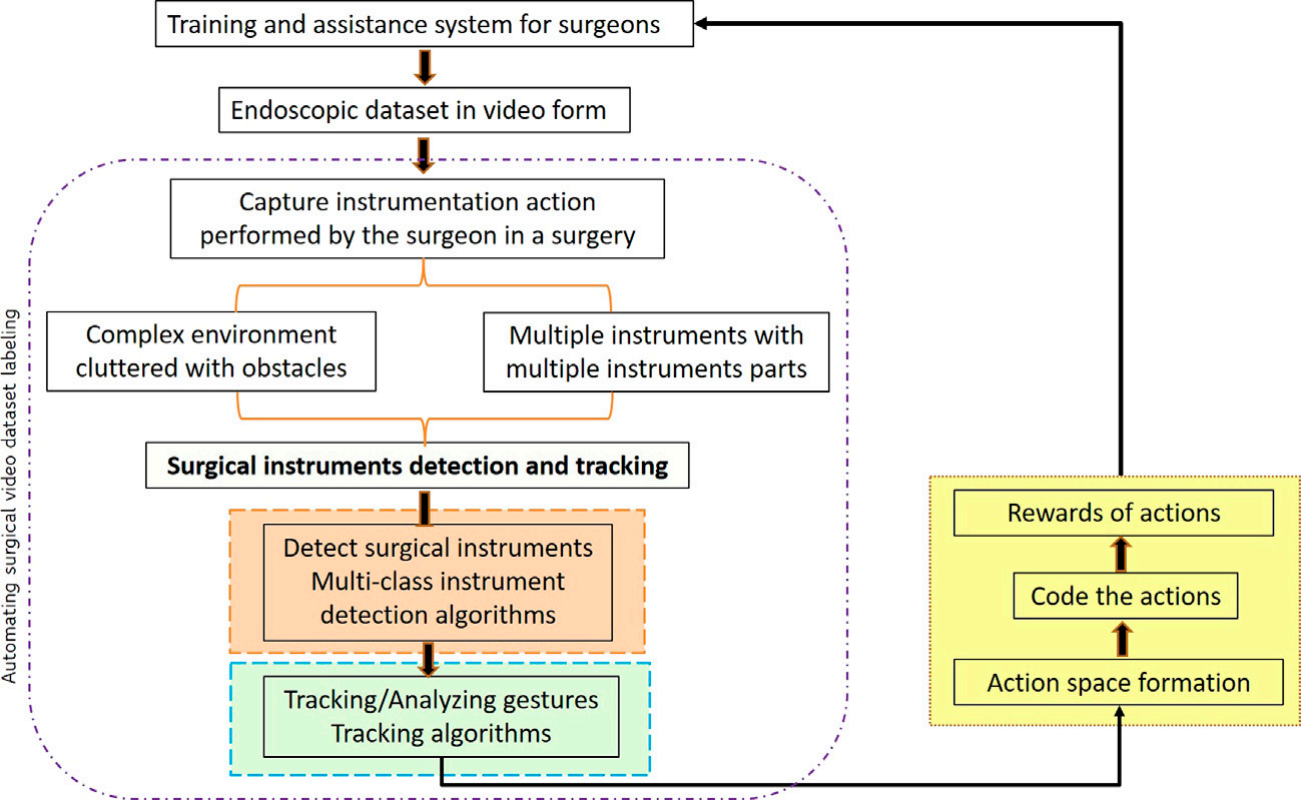
Various stages in the surgical skill assessment approach for a training or assistance system (the basis for the categorization of existing work).

The intraoperative and post-operative impediments in surgical practice remain a clinical challenge. Procedure-related factors and the lack of technical skills of a surgeon sometimes increase the risk of adverse surgical outcomes (Fecso et al., 2019). A study involving 20 bariatric surgeons was conducted in Michigan, and it was observed that the technical skill of practicing bariatric surgeons varied widely, and better surgical skill with peer rating of operative skill leads to decreased postoperative complications (Birkmeyer et al., 2013). Therefore, in order to enhance patient outcomes, it is imperative to regularly provide objective feedback on the technical performance of surgeons. Compared with traditional open surgery, minimally invasive surgery (MIS) results in minor trauma, less bleeding, and faster recovery of the patient (Zhao et al., 2017). The surgeon’s skill level and the cooperation between the surgeons are key to this procedure (Rosen et al., 2011). To address the surgical skill assessment platform, existing works are placed in various stages of a typical learning method in Figure 1, which are as follows: first, a surgical scene is captured using an endoscope in the form of videos. These videos contain multiple instruments with different parts (the shaft and the metallic clasper or end effector) and varied and complex backgrounds. Second, surgical instruments used in a surgical intervention are to be tracked and detected. This procedure is a two-step procedure: 1) the detection or segmentation algorithm is trained to identify and localize surgical instruments (as shown in the orange box). Segmentation and detection algorithms are used to solve this purpose. 2) Motion features are extracted from the detected instrument localization throughout the video (as shown in the green box). The tracking of the instrument’s location from frame to frame can be initialized using learning algorithms. The two abovementioned steps are used to automatically generate labeling for a raw surgical video dataset. These dataset labelings comprise detected surgical instruments and motion features extracted from the tracking algorithm during an intervention. Third (as shown in the yellow box), an action space is generated based on the labeling to predict surgical skills, avoiding entirely simulator-based assessments. The action space comprises the locations of bounding boxes containing the shaft and the end effector captured during the movement of the surgical instrument and the rotation angle that the shaft made with the reference frame and the end effector made with respect to the shaft. The actions identified are then coded in order to generate rewards. These rewards are quantified to predict the surgical skill level of an individual surgeon.

The actions can be used by trainer surgeons to train newly budding surgeons in this field and used as an intraoperative assistance system to perform a surgical intervention. This three-stage modeling approach can be used to generate rewards and can help in distinguishing good versus poor surgical skills. While the degree of surgical skill cannot yet be reliably quantified using the technique represented, it represents an important advance toward the automation of surgical skill assessment. It is clear that a detection and tracking system is required to systematically quantify surgical skills for better assessment and training systems for laparoscopic surgeons. However, the challenge lies in dealing with complex operation scenes that are captured using endoscopes wherein raw surgical images have occlusions, i.e. blurry patches due to smoke, a variety of tissues, organs, etc.(Jin et al., 2020). In these complex scenarios, video processing for segmentation, feature identification, and tracking needs special attention. The study of a detection and tracking algorithm [based on artificial intelligence (AI) and machine learning (ML)] that meets the requirements of real-time accuracy and robustness for the development of surgical robots is an important step (Rosen et al., 2011).

In this review, we focus on the research contributions in the field of surgical instrument detection and tracking using AI methods. We will review some existing instrument detection and tracking algorithms in Section 2. Section 2 also presents a general overview of vision-based AI methods for instrument detection and tracking technology in minimally invasive surgical instruments in a classified manner from the perspective of “feature extraction” and “deep learning.” A comprehensive perspective of surveyed algorithms is discussed in Section 3, which aids in identifying the future directions for the development of vision-based AI algorithms to automate MIS skill assessment.

## 2 Vision-based AI techniques for surgical instrument detection and tracking

The state-of-the-art vision-based methods for detecting and tracking objects are well-developed. However, it is important to check if the environment and object (surgical tool in MIS and robot-assisted surgeries) combination is amenable to the existing vision-based algorithms. Therefore, this section provides critical and categorical discussions on relevant existing methods.

### 2.1 Overview

**FIGURE 2:**
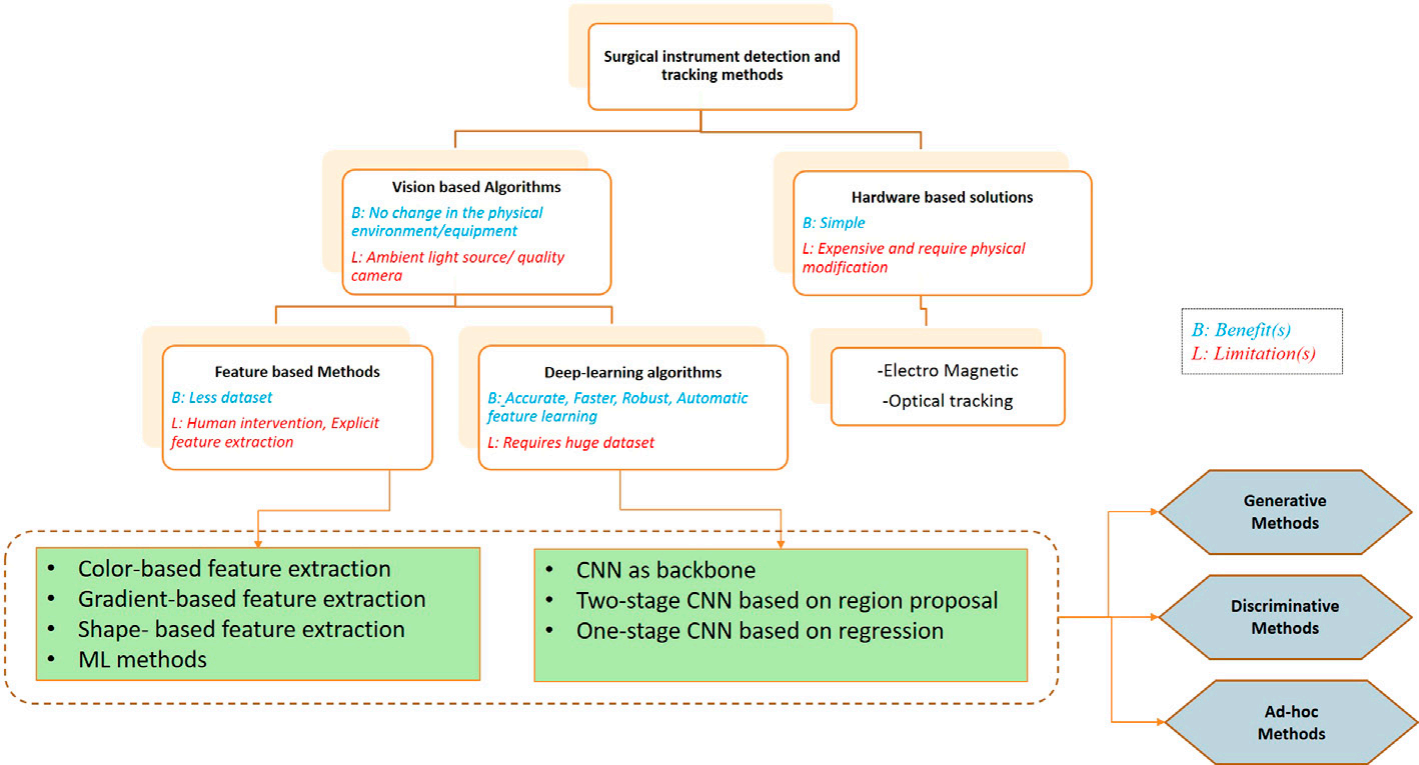
Categorization of methods for surgical instrument detection and tracking.

Vision-based AI surgical instrument detection and tracking technology in MIS combine machine vision, pattern recognition, deep learning, and clinical medicine (Wang et al., 2021). The overview graph describing the methods in this review is shown in Figure 2. Surgical instrument detection and tracking algorithms can be hardware-based or vision-based techniques. Surgical operation becomes cumbersome since hardware-based algorithms, despite their apparent simplicity, demand expensive hardware equipment and physical changes to the surgical setup (Choi et al., 2009; Schulze et al., 2010). Methods involve placing markers and trackers in order to track surgical instrument location (Krieg, 1993; Trejos et al., 2008; Yamaguchi et al., 2011). However, these existing devices are expensive; therefore, they are popular only in a limited number of medical centers and research institutes. The studies are concentrated on vision-based exploration, classified as feature-based methods and deep learning algorithms for surgical tool recognition and tracking as a result of the advancement of machine vision technology. These algorithms can be generative, discriminative, or ad hoc methods (Bouget et al., 2017; Wang et al., 2021). The generative method is typically based on estimating probabilities, modeling data points, and distinguishing between classes based on these probabilities. Discriminative methods, on the other hand, refer to a class of methods used in statistical classification, especially in supervised machine learning. They are also known as conditional models. Generative modeling learns the boundary between classes or labels in a dataset. It models the joint probability of data points and can create new instances using probability estimates and maximum likelihood. Discriminative models have the advantage of being more robust to outliers than generative models. Some examples of discriminative models are logistic regression, support vector machine (SVM), decision tree, and random forest. Ad hoc methods use low-level image processing techniques, such as thresholding. In the generative model, some methods of the discriminant model, such as SVM and random forest, require manual feature extraction, thus restricting the construction of high-level semantic information, and are not suitable for surgical instrument detection and tracking in complex environments (Rieke et al., 2016). Deep learning can express more advanced and finer details and semantic information without extracting the feature information explicitly (Twinanda et al., 2016; Alshirbaji et al., 2020). Therefore, deep-learning-based approaches are widely used and have become the mainstream research direction (Sahu et al., 2016; Garcia-Peraza-Herrera et al., 2017).

### 2.2 Feature-based methods

Most of the feature extraction methods focus on tracking the surgical instrument tip. Tip tracking is based on features like color, gradient, and shape.

#### 2.2.1 Color-based feature extraction

The color feature is most common for performing any operation in image processing. In 1994, Lee et al. (1994) used RGB color space in the detection of surgical instruments. Later on, HSV color space was developed (Doignon et al., 2004) for the surgical instrument tip based on its color and brightness from the surrounding environment. This method proved to be more robust to the lighting changes by decoupling the luminosity from other components. For tracking the surgical instruments, Wei et al. (1997) developed a stereoscopic laparoscopic vision tracking method where color marking is tracked using the thresholding method of segmentation. This method was used to locate the instrument accurately and control the movement. However, for complete detection and tracking tasks, color space analysis was used (Allan et al., 2012). Later, in 2006, Tonet et al. (2007) applied the image processing method to endoscopic images. A colored strip with auxiliary markers is added to the surgical instrument to facilitate segmentation. This helps the automatic tracking of surgical instruments without interfering with the actual surgical settings and without any pre-operative procedures. This method works well in scenarios where low precision is required, such as surgical assistance tasks at initial levels (Wang et al., 2021).

#### 2.2.2 Gradient-based feature extraction

Instrument detection and tracking based on the color feature are affected by the illuminating light reflected from the metal surface. Thus, to solve this problem, Pezzementi et al. (2009) used the gradient feature, which depends on the intensity values and specific color space component of images. However, it requires large marker data to visualize the appearance change of the surgical instrument. In 2013, depth maps were developed as an effective way for gradient calculation with different types of images as inputs (Haase et al., 2013). The Sobel operator is used along with the histogram of oriented gradients (HOGs) to estimate the edge information probability to detect the edge of a surgical instrument. In 2015, Rieke et al. (2015) used the HOG to perform surgical tool detection. They performed detection and tracking for three sets of instrument joints in a metallic clasper. Their method gives correct position estimates of the surgical instrument in challenging backgrounds cluttered with lighting and noise.

#### 2.2.3 Shape-based feature extraction

Shape-based feature extraction was also introduced for surgical instrument detection that expressed as a set of generated numbers. Otsu’s based method was used by Voros et al. (2007) to identify the tip position of the instrument. For this, the cumulative moments are calculated to determine the optimal distance between the surgical tool and the backdrop pixels. This approach is limited to the presence of noise, occlusion, or non-rigid deformation, but is scale-invariant under complex transformations, including translation, scaling, and rotation. Bayesian filters proved to be the most robust and effective feature extraction method for surgical instrument tracking in retinal microsurgery (Sznitman et al., 2012). These filters are trained to sum the pixel intensities within the boundary and respond strongly to the sharp directional intensity edge.

#### 2.2.4 ML methods

Before the advent of deep learning, ordinary learning algorithms were used in the visual detection and tracking of surgical instruments. These methods use the characteristic features of surgical instruments under different conditions to train a model. Traditional methods require extracting features from an image manually to detect and track surgical instruments from raw surgical images. These methods involve classical appearance-based machine learning methods and other discriminative machine learning methods. Models such as random forests (Bouget et al., 2015) and maximum likelihood Gaussian mixture models (Pezzementi et al., 2009) trained on color-based features have been applied. An approach based on appearance learning (Reiter et al., 2014) has been developed to detect shaft and specific points on the robotic surgical instrument tip using laparoscopic sequences. The algorithm can work in a highly dynamic environment and remain unchanged in light and posture changes.

### 2.3 Deep learning-based methods

With the increasing advancement of deep learning in various areas, such as image segmentation, natural language processing, image registration, and object tracking, deep learning has also become the main research direction in the visual detection and tracking of surgical instruments.

One of the most popular deep learning networks is convolutional neural networks having the ability to handle large amounts of data (Alzubaidi et al., 2021). The use of hidden layers has surpassed traditional techniques. Over the years, CNNs have become a crucial part of computer vision applications, especially pattern recognition. CNNs are a class of deep neural networks that use a special technique called convolution (a mathematical operation that specializes in processing data that have a grid-like topology, such as an image frame). For surgical instrument detection and tracking, CNNs play a vital role. A CNN typically has three layers: a convolutional layer, a pooling layer, and a fully connected layer (FC). This layer performs a dot product between the restricted portion of the receptive field of an image and a kernel matrix with a set of learnable parameters. The pooling layer replaces the output of the network at certain locations by deriving a summary statistic of the nearby outputs and thus reduces the amount of computation and weights. The FC layer helps map the representation between the input and the output. Since convolution is a linear operation and images are far from linear, non-linearity layers are often placed directly after the convolutional layer to introduce non-linearity to the activation map. There are several variants of CNNs utilized for various applications of MIS (Jaafari et al., 2021). A few that are used for surgical instrument detection and tracking are described in the following sections.

#### 2.3.1 CNN as the backbone network

Algorithms for surgical instrument detection and tracking in minimally invasive surgery were developed by García-Peraza-Herrera et al. (2016) in 2016. They created a fully convolutional network (FCN) incorporating optical flow for tracking. The technique is better suited for the sluggish video data set of minimally invasive surgery and achieves an absolute accuracy of 88.3%. A CNN is also combined with an SVM and hidden Markov model (HMM) to improve AlexNet (i.e., EndoNet) to solve the surgical instrument detection and tracking problem in minimally invasive surgery (Twinanda et al., 2016). They developed a three-stage methodology for surgical instrument presence detection and phase recognition. EndoNet extracts visual features from the training images and transmits them to the SVM and hierarchical HMM to perform tracking of surgical instruments. This method gives an accuracy of 81% for a plethora of complex datasets. For the continuous detection of surgical instruments, Alshirbaji et al. (2020) combined CNNs with two long- and short-term memory (LSTM) models. The experimental findings demonstrate the advantages of integrating spatial and temporal data to create a robust and efficient technique to detect surgical instruments in raw laparoscopic videos. With a mean average precision of 91%, this technique outperforms EndoNet. Trump’s TernausNet-16, a deep convolutional neural network-based instrument tracking, was implemented in 2021 by Cheng et al. (2021). It is an extension of U-Net (Ronneberger et al., 2015) and constructed on VGG-16 (Simonyan and Zisserman, 2014). It combines deep neural network detectors and motion controllers and is based on a visual servo control framework. The network gathers visual data and determines the object’s location. Utilizing a kinematic controller, the visual content information extracted is used to calculate the joint velocities of the surgical robot. The cycle then continues with the revised image being sent to the network again. As a result, the system automatically tracks the target instrument to the center of the field of view. The TernausNet-16 network has demonstrated real-time surgical instrument detection in experiments, and magnetic endoscopes achieve accurate tracking.

Kurmann et al. (2017) presented a novel method for 2D vision-based recognition and pose estimation of surgical instruments that generalizes to different surgical applications. Their CNN model simultaneously recognizes the multiple instruments and their parts in the surgical scene. The network produces probabilistic outputs for both the presence of different instruments and the position of their joints. The parameters are optimized using cross-entropy loss. It is worth noting that their approach is parameter-free during test time and achieves good performance for instrument detection and tracking. They tested their approach on *in vivo* retinal microsurgery image data and *ex vivo* laparoscopic sequences. Hasan et al. (2021) improved upon the previous network by integrating CNN and algebraic geometry for surgical tool detection, segmentation, and estimating 3D pose. They developed Augmented Reality Tool Network (ART- Net), which uses deep separable convolution and global average pooling. The architecture is a single input–multiple output architecture which is more feasible than the general lightweight model. The average precision and accuracy of ART-Net reached 100%. Algorithms of two-stage CNNs based on region proposals are developed for surgical instrument tracking (Jin et al., 2018; Zhang et al., 2020). Compared with a separate CNN network, integrating multiple tasks into a single network can improve the accuracy of detection.

#### 2.3.2 Two-stage CNN: Region proposal-based algorithms

A region proposal network (RPN) and a multi-modal two-stream convolutional network for surgical instrument detection were developed by Sarikaya et al. (2017). A combination of image and temporal motion cues jointly performs object detection and localization task. The work introduced a new dataset performing six different surgical tasks on the da Vinci Surgical System with annotations of robotic tools per frame. A new modular anchor network based on faster R-CNN (Zhang et al., 2020) is developed to detect laparoscopic surgical instruments, which consists of using a relationship module and an anchor generation mechanism. The proposed two-stage method generated adaptive shape anchors to detect instruments using semantic information. The range of deformable convolution is expanded based on a modulation feature module. On the new private data set (AJU-Set) and the public data set (m2cai16-tool-locations), their method yields detection accuracies of 69.6% and 76.5%, respectively. A CNN cascade for real-time surgical instrument detection (Zhao et al., 2019) has been developed to use in robot-assisted surgery. The frame-by-frame instrument detection is carried out by cascading two different CNN networks. An hourglass network that outputs detection heatmaps for tool-tip area representation and a modified visual geometry group (VGG) network for creating a bounding box around the detected part are developed. These two networks jointly predict the localization. The method is evaluated on the publicly available EndoVis Challenge dataset, and the ATLAS Dione dataset and achieves good accuracy in terms of speed and performance.

#### 2.3.3 One-stage CNN: Regression-based algorithms

Although algorithms of two-stage CNNs based on region proposal have a higher accuracy, the real-time performance of most algorithms is still slightly inferior to that of the one-stage algorithm. In 2017, a one-stage CNN algorithm based on regression (Choi et al., 2017) was developed to achieve minimally invasive surgical instrument detection and tracking task. The algorithm has been introduced as a simple regression problem. The CNN model developed could detect and track surgical instruments in real time with a reduced number of parameters through down-sampling. In 2020, Wang and Wang (2020) proposed a one-stage instrument detection framework controlled by reinforcement learning. The proportion of positive training samples for the reinforcement learning framework is achieved by optimizing the negative sample candidate frame and thus improves the instrument detection model’s accuracy. The one-stage instrument detection method has an advantage over the two-stage detection method in terms of high-speed framework and accuracy. Compared with previous methods, this framework detects surgical tools with complex backgrounds with small training sample sizes. In 2021, Shi et al. (2020) developed a convolutional neural network enhanced with real-time attention guidance for frame-by-frame detection of surgical instruments in MIS videos. The method was verified on varied datasets with backgrounds having blur, occlusions, and deformations.

### 2.4 Toward surgical skill assessment

Researchers use the detection and tracking outcomes for surgical skill assessment. Funke et al. (2019) developed a deep learning-based video classification method for surgical skill assessment. They use an inflated 3D ConvNet to classify snippets extracted from a surgical video. During training, a temporal segment network is added to the network. They tested their methodology on the openly accessible JHU-ISI Gesture and Skill Assessment Working Set (JIGSAWS) dataset (a surgical activity dataset for human motion modeling). The data set consists of recordings of routine robotic surgical procedures carried out on a dry laboratory bench-top model. High skill categorization accuracy of between 95.1 and 100.0 percent is achieved by their method. Lavanchy et al. (2021) introduced a three-stage machine learning method to automate surgical skill assessment in laparoscopic cholecystectomy videos. This method trains a CNN to localize and detect the surgical instruments. The motion feature localization throughout time has been extracted in order to further train a linear regression model. This helps in predicting the surgical skills of a practitioner. The technique represents an important advance toward the automation of surgical skill assessment. However, it cannot reliably quantify the degree of surgical skill.

## 3 Discussion and conclusion

Robot-assisted minimally invasive surgery and surgical robotics applications now include the task of surgical instrument detection and tracking as standard procedures. In recent years, simulation has become an important tool for educating surgeons and maintaining patient safety. Simulation provides an immersive and realistic opportunity to learn technical skills (Agha and Fowler, 2015). Simulation is a standardized and safe method for surgeon training and evaluation. AI methods allow observed demonstrations to be tracked in the MIS, thus generating a standardized range of actions. In addition to the development of laparoscopic and minimally invasive surgical techniques, the use of simulation for training has received increasing attention, and there is evidence that skills acquired in simulation can be applied to real clinical scenarios. Simulations allow trainees to make mistakes in order to ask “what if” questions without compromising patient safety. Virtual reality simulators are being used to help professionals plan complex surgeries and assess postoperative risks (Gaba et al., 2001). Using simulation in isolation from traditional teaching methods will furnish the surgeon in training with skills, but the best time and place to use such skills come only with experience used by AI models.

This study reviewed the latest technologies in surgical instrument detection and tracking that can automate labeling in raw surgical video datasets. Real-time automated surgical video analysis by faithful identification of labels and action space can facilitate feedback on surgical skill performance and help design automatic surgical interventions. Specific labels or information about surgical instruments, such as their position, orientation, and type, used at any time of intervention, can be retrieved by generating time information showing the use of instruments during the surgery. The existing algorithms and technologies still need to be improved in terms of accuracy and real-time visual detection and tracking of surgical instruments. Recent advances in artificial intelligence have shifted attention to automated surgical skill assessment, particularly in robotic interventions. Robotic surgeries have the advantage that kinematic data of instruments and video recordings are readily available from the console. Performance metrics are computed to predict skill levels and focus on robotic kinematics data. One study combined motion features extracted from video and some kinematic signals. A deep convolutional neural network can successfully decode skill information from raw motion profiles *via* end-to-end learning without the need for engineered features or carefully tuned gesture segmentation. However, there are still many outstanding technical challenges to be solved under environmental conditions such as occlusion, smoke, or blood.

In addition to increasing real-time performance and accuracy, the technology’s future development should concentrate on the following development trends:

• Despite the fact that many algorithms based on deep mastering CNN have been proposed, there are risks that frequently arise at some stage in the software process, such as occlusion, blood, and smoke. Consequently, enhancing robustness to improve the performance of algorithms for the detection and tracking of surgical instruments is still the focal point of studies.
• The foundation for developing the algorithm of visual detection and surgical instrument tracking is the quality of the surgical image. At the current level, the subsequent detection and tracking of minimally invasive surgical instruments are significantly impacted by image quality and the hassle of the complex environment, and improving the quality of image acquisition is not negligible.
• For complete supervision of the deep learning model, manually labeled data by knowledgeable doctors are crucial. However, many data labeling findings cannot be used because of insufficient and faulty labeling and the collection of datasets, leaving many training samples devoid of valuable markers. When employing automated visual data labeling to detect and track surgical instruments, weak or unsupervised deep learning models can improve the algorithm’s accuracy and real-time performance.
• Only when complete and publicly available verification data sets and methods are available, the effectiveness and progress of surgical instrument detection and tracking algorithms can be accurately measured.

This study compares the test results and application scope of various visual detection and tracking algorithms for surgical instruments. Some future development trends and directions of this technology for development toward an automated surgical skill assessment system are given so that researchers in related fields can have a more systematic understanding of the current research progress.

## Glossary

AI: artificial intelligence: a branch of computer science concerned with building machines capable of performing tasks as humans do
ML: machine learning: a branch of artificial intelligence (AI) and computer science which focuses on the use of data and algorithms to imitate the way that humans learn, gradually improving its accuracy
MIS: minimally invasive surgery: a new standard of care for surgical procedures
robot-assisted MIS: MIS with improved control and dexterity
feature extraction: the process of transforming raw data into numerical features that can be processed while preserving the information in the original data set
deep learning: a type of machine learning and artificial intelligence (AI) that imitates the way humans gain certain types of knowledge
segmentation: the process of dividing an image into groups of similar pixel intensity;
SVM: a type of deep learning algorithm that performs supervised learning for classification or regression of data groups
CNN: a type of artificial neural network used in image recognition and processing that is specifically designed to process pixel data
RGB color space: an electronic way to represent images
HOG: histogram of oriented gradients: a feature descriptor used in computer vision and image processing for object detection;
supervised and unsupervised learning: supervised learning uses labeled input and output data, while an unsupervised learning algorithm does not
FCN: fully convolutional network: an architecture used mainly for semantic segmentation made up of convolutional layers. HMM, hidden Markov model: a statistical Markov model in which the system being modeled is assumed to be a Markov process
LSTM: long short-term memory networks are a type of recurrent neural network capable of learning order dependence in sequence prediction problems
EndoNet: a type of deep learning architecture for recognition tasks
U-Neta: convolutional neural network that was developed for biomedical image segmentation at the Computer Science Department of the University of Freiburg
VGG-16: very deep convolutional networks for large-scale image recognition, a type of deep learning network developed by K. Simonyan and A. Zissermen
ART-Net: a type of deep learning network
3D-pose: articulated 3D joint locations of an object from an image or video
RNN: recurrent neural network, a network that recognizes sequential characteristics of data and uses patterns to predict the next likely scenario
regression: a technique for investigating the relationship between independent variables or features and a dependent variable or outcome
